# Editorial: Transplant International Goes for GOLD!

**DOI:** 10.3389/ti.2022.10340

**Published:** 2022-02-03

**Authors:** Maria Irene Bellini, Nuria Montserrat, Maarten Naesens, Thomas Neyens, Stefan Schneeberger, Thierry Berney

**Affiliations:** ^1^ Social Media Editor, Transplant International; ^2^ Deputy Editor-in-Chief, Transplant International; ^3^ Statistical Editor, Transplant International; ^4^ Editor-in-Chief, Transplant International

Transplant International is starting the New Year with a new publisher. After a rigorous review process, Frontiers Partnerships was considered the best fit four our mission and was selected based on their high quality performance and their enthusiasm for active engagement in our journal (1,2).

One key criterion was our determination to move to a Gold Open Access model. The publication of medical science in open access format has been growing over the past decade, on what is a seemingly irreversible path. Indeed, in 2020, the number of papers published in open access exceeded for the first time those accessible by subscription only (3). We believe that open access publication is part of the dynamic process of open research, which starts with the publication of research in pre-print servers while the manuscript undergoes revisions and improvements, and ends with the granting of full access to source data, for the sake of transparency, reproducibility of experiments, and the fostering of more rigorous science. These considerations are embodied in the FAIR guiding principles for scientific data management - Findability, Accessibility, Interoperability, and Reusability- (4,5) to which Transplant International explicitly adheres.

Open access publishing has become a general request from academic institutions to their scholars, but also, and more compellingly, from most funding agencies who require that all scientific outputs resulting from their grants be made freely available to all (6,7). Open access publishing cannot exist without payment of an author publication fee, which may sometimes generate some frustration, but is covered by an increasing number of funding agencies and institutions.

However, there is much more to open access than the ethics of open science, institutional requests or publication costs. The common goal for all stakeholders in the scientific publication process is to disseminate research and increase its overall quality, acknowledgment, visibility and notoriety. Depending on perspective, a variety of metrics are available. They are useful benchmarking indicators, designed to measure different types of impact. Although often confused, they are not interchangeable. The Impact Factor (IF) is an indicator of where a specific journal is standing in the landscape of scientific titles, but not of a particular article or author. Usage metrics for articles (downloads, views, engagements and captures) or the h-index for authors were designed for this purpose (8). Most of these metrics are driven by citations in the scientific literature and will increase through a higher rate of citations.

Evidence indicates that open access publication confers a citation advantage, at least in selected fields of medical science (9-13). The citation advantage is beneficial to all parties involved, and in particular the authors, but also the academic institutions (14) and the journal (15,16).

Social media have emerged to a leading position among the tools of fast dissemination of scientific output (17,18). They are widely used for this purpose by investigators, but also by social media editors of scientific journals (18). Alternative metric scores (such as Altmetric or PlumX) comprehensively assess usage, captures, mentions, social media posts and citations, but also new categories -such as clinical or policy citations, news articles or mentions, blog posts, comments, reviews or links-that indicate active engagement and repetitive interactions with the public (8). They give an interesting idea of the immediate “social” attention gained by a particular scientific publication, as opposed to the h-index which takes years to build for a particular author (8). They measure its impact in the web community, and very often positively influence its future citations (13). There is in fact growing evidence for a link between citations, altmetric scores and open access, at least in certain fields of biomedical research (18-20).

Transplant International is starting this year with a lot of ambitions (1,2), that we believe will be better served by our choice to go for gold. We are confident that our readership, but also the authors submitting their valuable work to Transplant International, will embrace this choice and, as we thank you for your trust, we offer you our best wishes for a successful 2022. Happy New Year !
